# *Mulloidichthys flavolineatus flavicaudus* Fernandez-Silva & Randall (Perciformes, Mullidae), a new subspecies of goatfish from the Red Sea and Arabian Sea

**DOI:** 10.3897/zookeys.605.8060

**Published:** 2016-07-14

**Authors:** Iria Fernandez-Silva, John E. Randall, Daniel Golani, Sergey V. Bogorodsky

**Affiliations:** 1 Section of Ichthyology, California Academy of Sciences, 55 Music Concourse Dr, San Francisco, CA 94118, U.S.A.; 2 Departament of Biochemistry, Genetics and Immunology, Campus Universitario, University of Vigo, 36310 Vigo, Spain; 3 Bishop Museum, Honolulu, 1525 Bernice Street, Honolulu, HI 96817, U.S.A.; 4 Department of Evolution, Systematics and Ecology, the Hebrew University of Jerusalem, Israel; 5 Senckenberg Research Institute and Natural History Museum Frankfurt, Senckenberganlage 25, 60325 Frankfurt a.M., Germany; 6 Station of Naturalists, Tulenina Str 13-29, Omsk, Russia

**Keywords:** cytb, marine fish, glacial refugia, phylogeography, taxonomy, vicariance

## Abstract

The number of goatfish species has increased recently, thanks in part to the application of molecular approaches to the taxonomy of a family with conservative morphology and widespread intraspecific color variation. A new subspecies *Mulloidichthys flavolineatus flavicaudus* Fernandez-Silva & Randall is described from the Red Sea and Arabian Sea, including Socotra and Gulf of Oman. It is characterized by a yellow caudal fin, 25–28 gill rakers, and 37–38 lateral-line scales and it is differentiated from nominal subspecies *Mulloidichthys flavolineatus flavolineatus* by 1.7% sequence divergence at the mitochondrial cytochrome b gene. The morphometric examination of specimens of *Mulloidichthys flavolineatus flavolineatus* revealed variation in head length, eye diameter, and barbel length, in western direction from the Hawaiian Islands, South Pacific, Micronesia, and the East Indies to the Indian Ocean. The population of *Mulloidichthys flavolineatus flavicaudus* **subsp. n.** in the Gulf of Aqaba differs from that of the remaining Red Sea by shorter barbels, smaller eyes, shorter head, and shorter pelvic fins. We present a list of 26 endemic fishes from the Gulf of Aqaba and discuss the probable basis for the endemism in the light of the geological history of this region.

## Introduction

**Figure 1.**
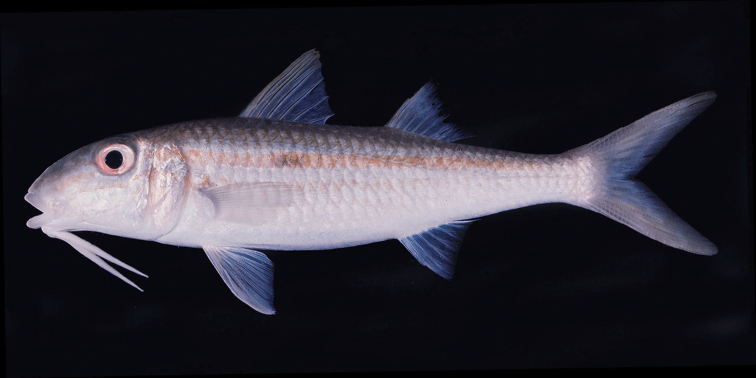
Color photograph of the neotype of *Mulloidichthys flavolineatus flavolineatus* from Mauritius, BPBM 20135, 162 mm SL. Photo by John E. Randall.

**Figure 2.**
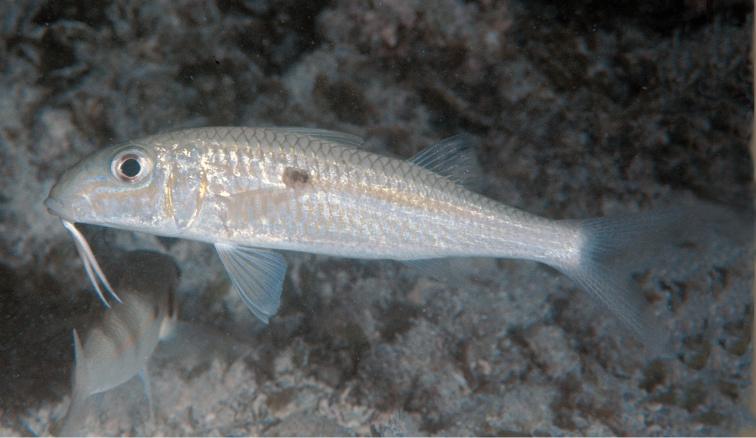
Underwater photograph of *Mulloidichthys flavolineatus flavolineatus* (aprox. 230 mm SL) from Mauritius, the type locality of the subspecies. Photo by John E. Randall.

The goatfish *Mulloidichthys flavolineatus* was described by Lacepède (1801) based on a manuscript written by Dr. Philibert Commerçon (Commerson in English). There is no type specimen and no record of the type locality (Bauchot et al. 1985). It is almost certainly Mauritius, where Commerson spent several years collecting biological specimens, including many fishes. Fricke (1999: 309) designated a neotype for *Mulloidichthys flavolineatus* from nearby Réunion, but it was later considered invalid by him (Fricke 2000: 639) as “not sufficiently in accordance with Article 75b and Article 75d of the International Code of Zoological Nomenclature.” We designate and describe a neotype in the present paper (Fig. 1) collected and photographed in Mauritius by the second author. We also illustrate a live individual from the island (Fig. 2).

*Mulloidichthys flavolineatus* is presently regarded as the most wide-ranging species of the family Mullidae, from the northern Red Sea (Ben-Tuvia and Kissil 1988) to the Pitcairn Islands (Nichols 1923; Randall 1999). Such a broad distribution might be expected from the unusually large size attained by the postlarvae at settlement, 60 to 80 mm SL (Randall 2005). It is also unusual for such a common and widespread species to have only two junior synonyms, *Mulloides samoensis* Günther, 1874, type locality, Upolu, Samoa Islands, and *Upeneus preorbitalis* Smith & Swain, 1882, type locality, Johnston Atoll.

**Figure 3.**
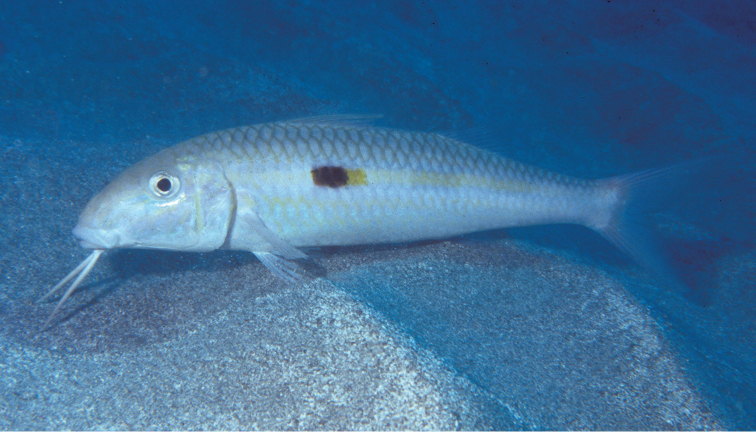
Underwater photography of *Mulloidichthys flavolineatus flavolineatus* from O‘ahu, Hawai‘i. Photo by John E. Randall.

**Figure 4.**
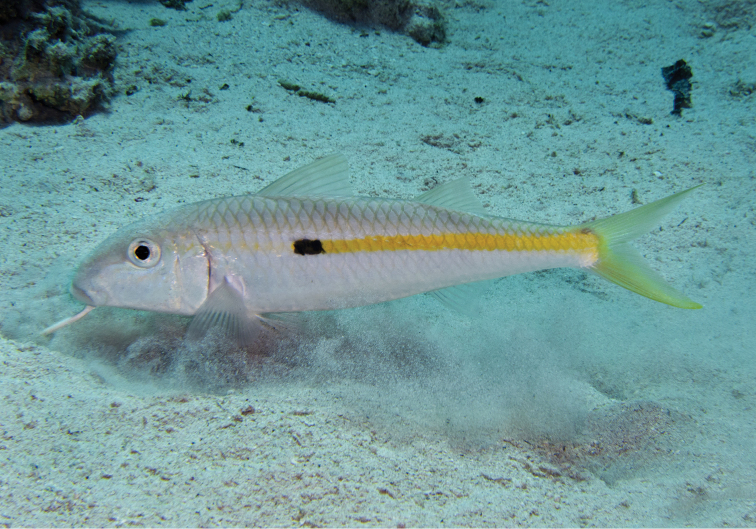
Underwater photographs of *Mulloidichthys flavolineatus flavicaudus* subsp. n. from Dahab in the Gulf of Aqaba. Photo by Sergey V. Bogorodsky.

**Figure 5.**
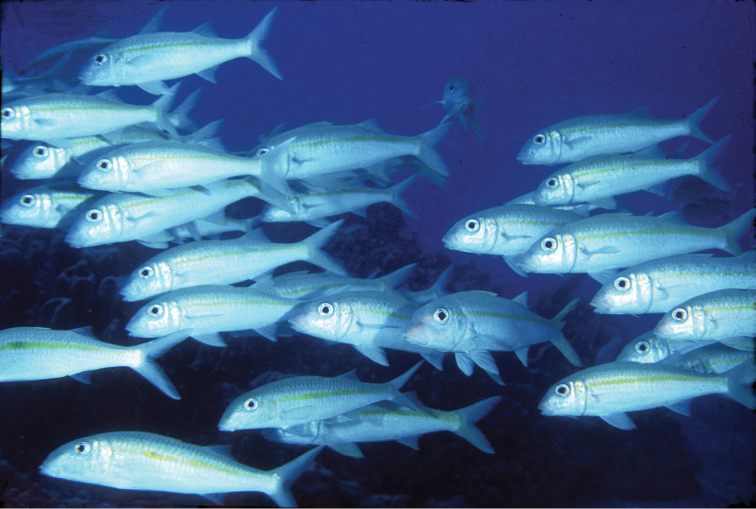
School of *Mulloidichthys flavolineatus flavolineatus* in Maui, Hawai‘i. Photo by John E. Randall.

**Figure 6.**
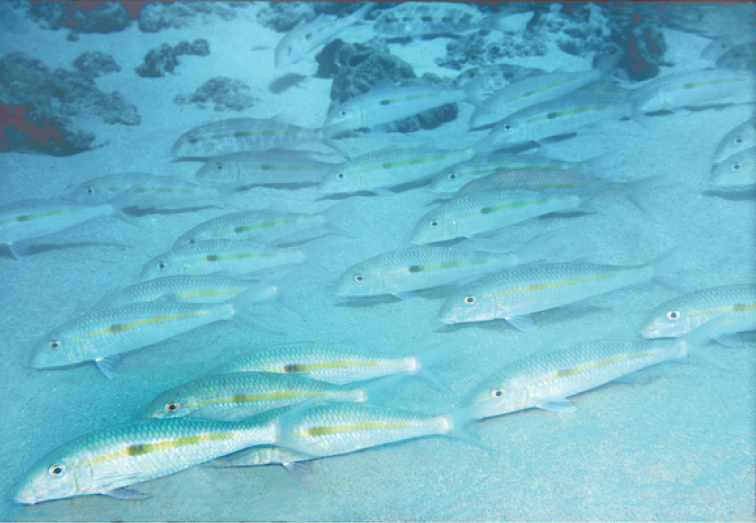
School of *Mulloidichthys flavolineatus flavolineatus* in Maui, Hawai‘i resting on the bottom. Photo by John E. Randall.

Like other goatfishes, this species uses the pair of sensory barbels on its chin to locate prey, mainly in sedimentary substrata, as seen in Fig. 3 of an adult in the Hawaiian Islands and one in the Red Sea (Fig. 4). Randall (2005: 292) summarized the prey of specimens from the Hawaiian Islands as small crabs, shrimps, polychaete worms, small bivalve mollusks, hermit crabs, crab megalops, heart urchins, small gastropods, amphipods, foraminifera, and unidentified eggs. During periods of inactivity, the fish may be seen hovering in aggregations a short distance above the bottom (Fig. 5) or in groups resting on sand (Fig. 6).

Myers (1999: 159) reported spawning in Palau over shallow sandy areas near the reef’s edge for several days following new moon. Females in the Mariana Islands may be mature as small as 123 mm in SL, and males as small as 112 mm. The spawning season is December to September, with peaks from March to April. Large aggregations of silvery postlarvae settle out between March and June to shallow water on reef flats where they are often caught in seines or throw nets.

**Figure 7.**
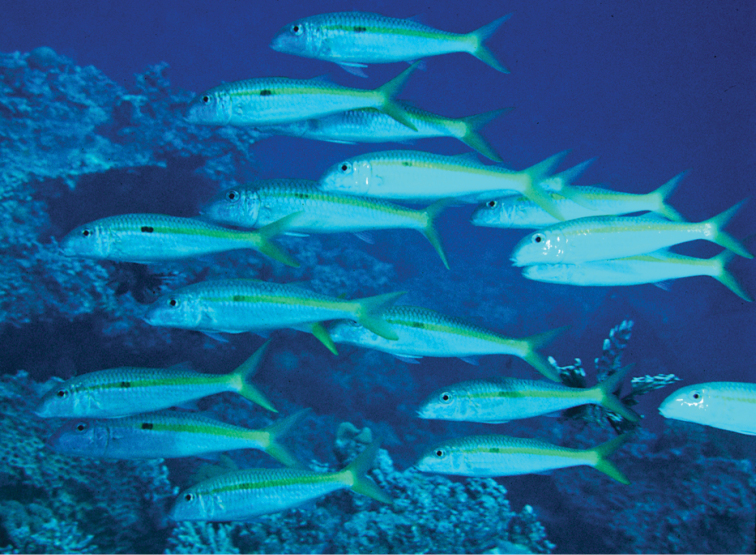
School of *Mulloidichthys flavolineatus flavicaudus* subsp. n. at Eilat, Gulf of Aqaba. Photo by John E. Randall.

**Figure 8.**
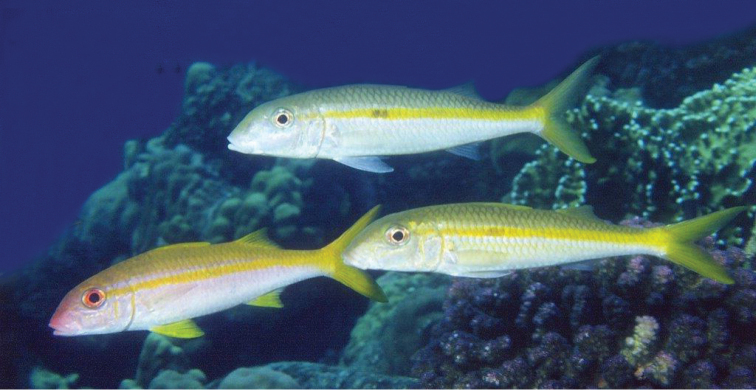
Underwater photography of two *Mulloidichthys flavolineatus flavicaudus* subsp. n. and one *Mulloidichthys vanicolensis* (left) in the Saudi Red Sea off Jeddah. Photo by Hagen Schmid.

We, and surely others, have noticed that the population of *Mulloidichthys flavolineatus* in the Red Sea has only yellow caudal fin (Fig. 7), whereas in most of the Indian Ocean and in the Pacific, the caudal fin is usually gray but occasionally also yellow. This goatfish should not be confused with *Mulloidichthys vanicolensis* (Valenciennes, 1831), which also has a yellow caudal fin (lead fish of the three of Fig. 8), as well as yellow dorsal, anal, and pelvic fins, whereas pelvic and dorsal fins are whitish in *Mulloidichthys flavolineatus*. The geographic distribution of the two color morphs of *Mulloidichthys flavolineatus* matches the distribution of two distinct mitochondrial lineages with 1.7% divergence at the cytochrome b (*cytb*) gene (Fernandez-Silva et al. 2015).

**Figure 9.**
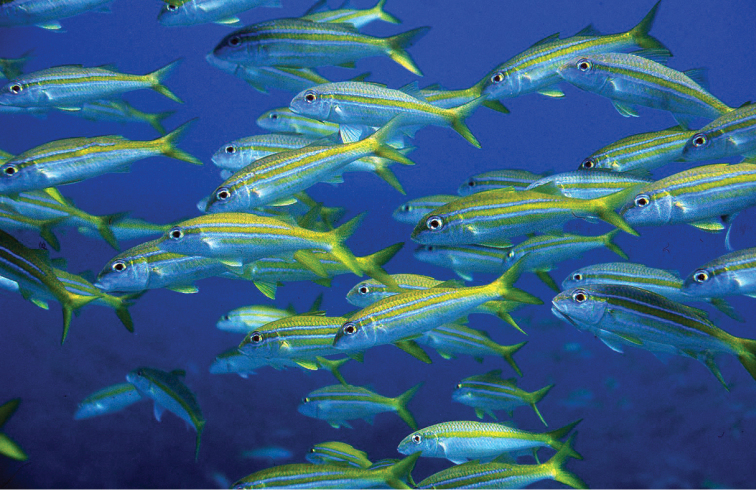
*Mulloidichthys ayliffe* with one individual of *Mulloidichthys flavolineatus flavicaudus* subsp. n. at Socotra. Photo by Hajnalka Kovacs.

The caudal fin continues to be yellow from the Red Sea into the Gulf of Aden and Socotra, as shown by Fig. 9, where a few individuals of *Mulloidichthys flavolineatus* have mixed with a school of *Mulloidichthys ayliffe*. Uiblein (2011) described the latter in a review of the species of *Mulloidichthys* of the Western Indian Ocean. It mimics and often schools with the snapper *Lutjanus kasmira*. It is an amazing example of parallel evolution with *Mulloidichthys mimicus* Randall & Guézé, 1980 of the Marquesas Islands and Line Islands in the Central Pacific, which closely mimics the stripe pattern of *Lutjanus kasmira* and forms aggregations with it.

**Figure 10.**
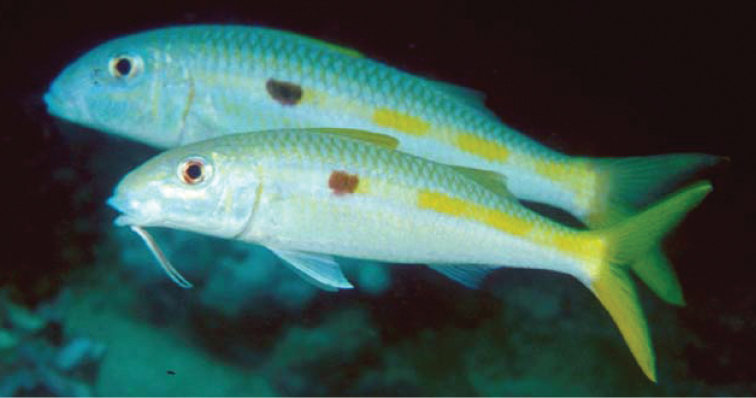
*Mulloidichthys flavolineatus flavicaudus* subsp. n. in Fahal Island in the Gulf of Oman. Photo by Richard Field.

**Figure 11.**
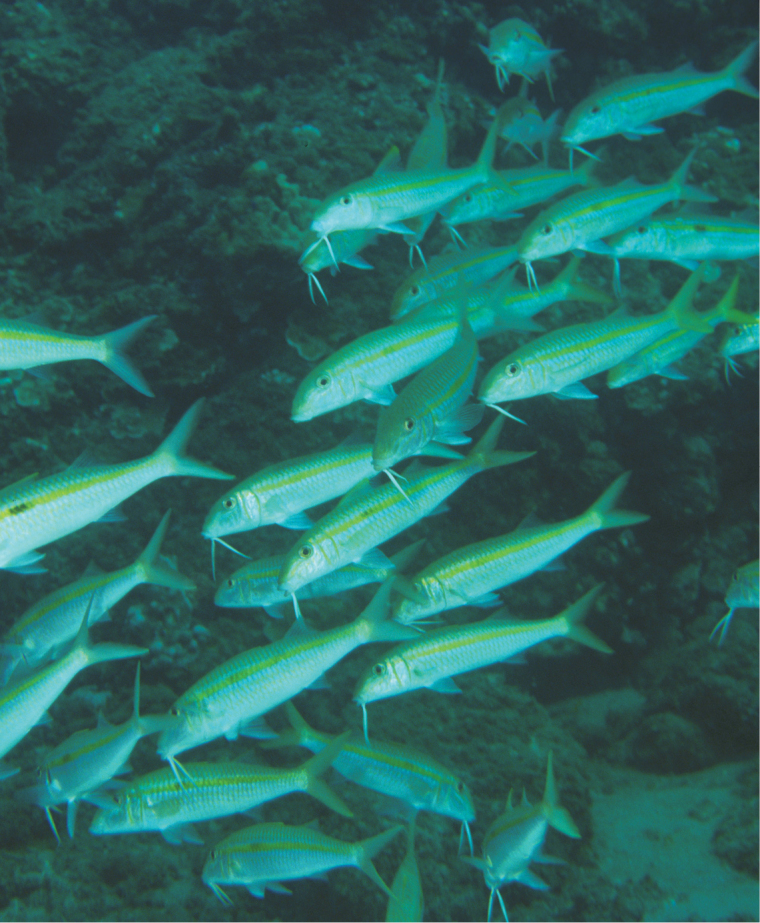
School of *Mulloidichthys flavolineatus* in Oman, some fish with white caudal fins and some fish with yellow caudal fins. Photo by John E. Randall.

**Figure 12.**
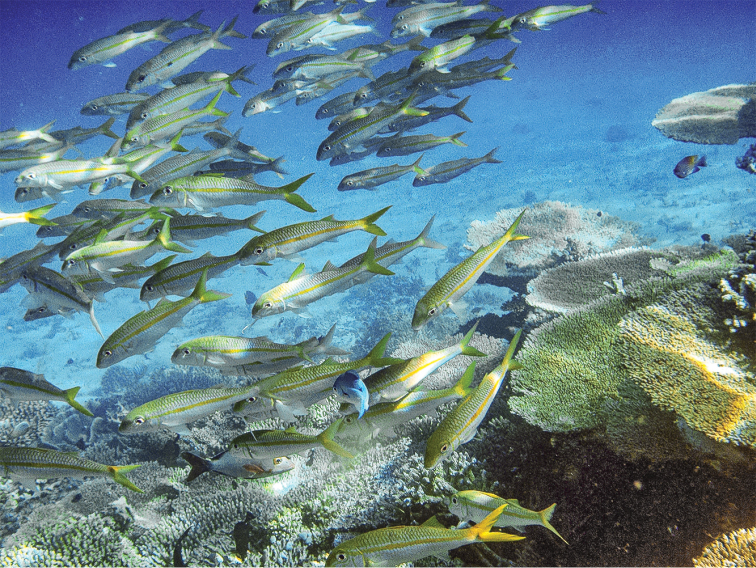
School of *Mulloidichthys flavolineatus* in South Ari Atoll in the Maldives, with some fish with whitish caudal fins in the background and other fish with caudal fins with different shades of yellow in the front. Photo by Rainer Kretzberg.

Across the Arabian Sea to the south coast of Oman aggregations of *Mulloidichthys flavolineatus* in Oman and Maldives include many individuals with yellowish caudal fin mixed with a few gray-tailed and yellow-tailed fish (Figs 10, 11 and 12). Elsewhere, caudal fins are predominantly white or light gray, although we have observed that the color of the caudal fin in individuals from South Africa to French Polynesia and Hawaiian Islands may vary from hyaline gray (predominantly) to yellow (occasionally).

## Methods

### Measurements and counts

Type specimens were deposited at the Bernice P. Bishop Museum, Honolulu, HI, U.S.A. (BPBM); the California Academy of Sciences, San Francisco, CA, U.S.A. (CAS); the Museum of the Hebrew University of Jerusalem, Israel (HUJ); the Senckenberg Museum, Frankfurt, Germany (SMF); and the U.S. National Museum of Natural History
(NMNH). These were the primary sources of goatfish specimens examined in this study.

Lateral-line counts begin with the first pored scale completely posterior to the upper end of the gill opening and end at the base of the caudal fin (three pored scales continue onto the caudal fin). Counts of gill rakers were made on the first gill arch; they include all rudiments.

Lengths of specimens are given as standard length (SL), measured from the median anterior point of the upper lip to the base of the caudal fin (posterior end of the hypural plate); body depth is taken vertically from the base of the first dorsal-fin spine where it emerges from the body (not the internal base); body width is the maximum width measured just posterior to the gill openings; head length (HL) from the front of the upper lip to the posterior end of the opercular membrane, and snout length from the same anterior point to the nearest fleshy edge of the orbit; orbit diameter is the greatest fleshy diameter, and interorbital width the least fleshy width; upper-jaw length is taken from the front of the upper lip to the end of the maxilla; barbel length is the maximum straight length; caudal-peduncle depth is the least depth, and caudal-peduncle length the horizontal distance between verticals at the rear base of the anal fin and the caudal-fin base; length of fin spines and rays of the dorsal and anal fins are measured from where they emerge from the body to their tip; caudal-fin length is the horizontal length from the posterior end of the hypural plate to a vertical at the tip of the longest ray; caudal concavity is the horizontal distance between verticals at the tips of the shortest and longest rays; pectoral-fin length is measured from the base of the uppermost ray; pelvic-fin length is measured from the base of the pelvic spine to the tip of the longest soft ray. Proportional measurements in the text are rounded to the nearest 0.05.

Only meristic characters and measurements that vary between *Mulloidichthys flavolineatus flavolineatus* and *Mulloidichthys flavolineatus flavicaudus* subsp. n. were applied in the diagnoses and comparisons: the number of gill rakers, lateral-line scale counts, barbel length, eye diameter and head length. We also compared the length of the pectoral and pelvic fins, but these did not show differences between *Mulloidichthys flavolineatus flavolineatus* and *Mulloidichthys flavolineatus flavicaudus* subsp. n.

Because goatfishes present allometric changes in body form (Uiblein and Heemstra 2010) during ontogeny, in the current study we only included fish > 73 mm and <288 mm.

### Genetic methods

During a previous phylogeographic survey of *Mulloidichthys flavolineatus* we obtained *cytb* sequences from 217 specimens sampled at nineteen sites throughout the Red Sea, the Arabian Sea, the Indian Ocean and the Pacific Ocean. To elucidate phylogenetic relationships we sequenced an additional fragment of the mitochondrial genome, the ATP synthetase 8 and ATP synthetase 6 (ATPase-8 and ATPase-6) regions, from individuals representative of the *cytb* diversity. We also sequenced an individual of *Mulloidichthys vanicolensis* and one of *Mulloidichthys pfluegeri* to use as outgroups. Briefly, DNA was extracted from fin clips and Polymerase Chain Reactions (PCR) were carried out using the primers L8331 (5'-AAA GCR TYR GCC TTT TAA GC-3') and H9236 (5'-GTT AGT GGT CAK GGG CTT GGR TC-3') (Meyer 1993). We carried out PCRs in a 15 µl volume containing 5 to 20 ng of template DNA, 0.1 µM of each primer and 5 µl of BioMix Red™ (Bioline Inc., Springfield, NJ, U.S.A.) in deionized water. PCRs were carried out with an initial denaturation step of 95 °C for 4 min, 35 cycles of denaturation (95 °C for 30 s), annealing (52 °C for 30 s) and extension (72 °C for 45 s), followed by a final extension step of 72 °C for 10 min. To clean PCR products we treated them with 0.75 units of Exonuclease I and 0.5 units of Fast Alkaline Phosphatase (ExoFAP; Thermo Fisher Scientific, Waltham, MA, U.S.A.) per 7.5 µL of PCR product, at 37 °C for 15 min, followed by deactivation at 85 °C for 15 min. We cleaned all PCR products using ExoSAP (USB, Cleveland, Ohio) and then sequenced them in the forward direction (and reverse direction, where appropriate) using a genetic analyzer ABI 3130XL (Applied Biosystems, Foster City, California) at the Hawai‘i Institute of Marine Biology EPSCoR Sequencing Facility. The *ATPase-8* and *ATPase-6* sequences were aligned, edited, and trimmed to a common length using GENEIOUS PRO vers. 4.8.4 (Drummond et al. 2012), and the sequences were deposited in Genbank (accession numbers: KT960949–KT960972). We concatenated this alignment with the *cytb* sequences from the same specimens and applied Bayesian methods for phylogenetic reconstruction in BEAST *vers.* 1.8.0 (Drummond et al. 2012), based on Yule models of speciation and a strict molecular clock (1% per myr as per Bowen et al. 2001). We also applied Neighbor-joining distance and Maximum-Likelihood tree-building methods for phylogenetic reconstruction using MEGA (Tamura et al. 2013) and the RaxML web server at http://embnet.vital-it.ch/raxml-bb/ (Varsamos et al. 2005), respectively. Support for the trees was evaluated by bootstrapping over 1,000 replicates.

## Data resources

The data underpinning the analysis reported in this paper are deposited in the Dryad Data Repository at http://dx.doi.org/10.5061/dryad.f54m5

## Results

### Mulloidichthys flavolineatus flavicaudus

Taxon classificationAnimaliaPerciformesMullidae

Fernandez-Silva & Randall subsp. n.

http://zoobank.org/779C9D55-B037-4548-A717-F5C33BC1ACD5

Yellowtail Goatfish

**Figure 13.**
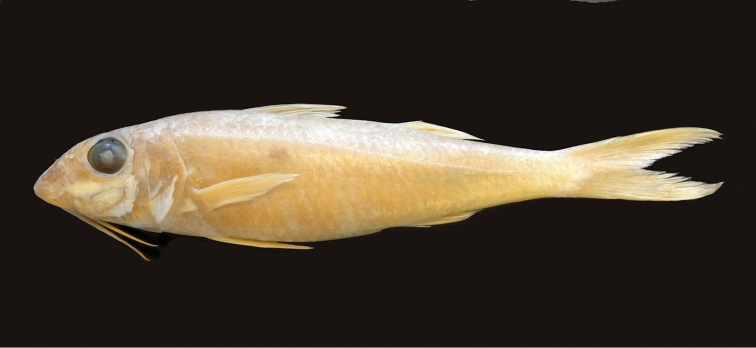
Preserved holotype of *Mulloidichthys flavolineatus flavicaudus* subsp. n., SMF 35486 [ex SMF 24824], 142 mm SL, Sanganeb Atoll, Sudan, Red Sea. Photo by John E. Randall.

Figures 4 , 7 , 8 , 9 , 10 , 13 (holotype), 14 ; Tables 1 , 2 , 3 , 4.

Mulloides flavolineatus (non Lacepède, 1801): Dor 1984: 161 (Red Sea listed); Ben-Tuvia and Kissil 1988: 3 (Gulf of Aqaba); Goren and Dor 1994: 44 (Red Sea listed); Debelius 1998: 112 (Egypt).
Mulloidichthys flavolineatus (non Lacepède, 1801): Randall 1995: 239 (Oman); Khalaf and Disi 1997: 117 (Jordan); Zajonz et al. 2000: 155 (Socotra); Lieske and Myers 2004: 123 (Mangrove Bay, El Quseir); Golani and Bogorodsky 2010: 35 (Red Sea listed); Field 2013: 47 (Gulf of Oman).

#### Holotype.

SMF 35486 [ex SMF 24824], 142 mm SL, Red Sea, Sudan, Sanganeb Atoll (19°39'N; 37°14'E), April 1991, coll. F. Krupp, V. Neumann & T. Paulus.

#### Paratypes.

SMF 24818, 6: 106–125 mm SL, Red Sea, Sudan, Sanganeb Atoll (19°39'N; 37°14'E), April 1991, coll. F. Krupp, V. Neumann & T. Paulus; USNM 221124, 181 mm SL, Red Sea, Egypt, off “ancient site” of Koseir along reef in cove ca. 5 km north of Koseir town (26°8'N; 34°16'E), 8 Jan 1965, coll. H. A. Fehlmann; HUJ 9985, 3: 73–93.5 mm SL, Red Sea, Gulf of Suez, El Kura, a few km south of Dahab (28°12'04"N; 34°28'49"E); CAS 237352, 4: 107–147 mm SL, Red Sea, Saudi Arabia, Thuwal, inner Fesar (22°13'50"N; 39°01'43"E), 18 April 2014, coll. P. Saenz-Agudelo; BPBM 41246 [ex CAS 237352], 102 mm SL, same data as preceding.

#### Non-type material.

**Gulf of Suez**: HUJ 5635, 107 mm SL, A-Tur. **Gulf of Aqaba**: BPBM 19843, 4: 114–122 mm SL, Nuweiba; CAS 58876, 252 mm SL, Eilat; CAS 206715, 133 mm SL, Eilat; CAS 206726, 198 mm SL, Eilat; CAS 206736, 167 mm SL, Eilat; HUJ 5905, 2: 128–144 mm SL, Eilat; HUJ 20216, 4: 107–110 mm SL (poor condition), Eilat; HUJ 8315, 169 mm SL, Ras Muhammed; HUJ 8543, 108 mm SL, Ras Muhammed; HUJ 8658, 2: 164–235 mm SL, Nabq; HUJ 8645, 3: 159–177 mm SL, Sanafir Island; HUJ 11663, 109 mm SL, Eilat; HUJ 8642, 243 mm SL, Nabek. **Sudan**: SMF 24821, 203 mm SL, Sanganeb Atoll; SMF 24823, 13: 97.5–161 mm SL, Sanganeb Atoll.

#### Diagnosis.

Body elongate, the depth at first dorsal-fin origin 4.1–4.5 in SL; head moderately compressed, the length 3.0–3.3 in SL; snout long, slightly blunt. Barbels usually not reaching a vertical at posterior margin of preopercle, their length 4.1-5.0 in SL. Eye diameter 10.3–13.5 in SL. Pectoral-fin rays 16–18. Gill-raker counts 25–28 (usually 26 or 27); lateral-line scales 37–38. Caudal fin yellowish to yellow. [Diagnosis based on the Red Sea proper population, i.e. excluding the Gulf of Aqaba, see remarks].

#### Description.

**Figure 14.**
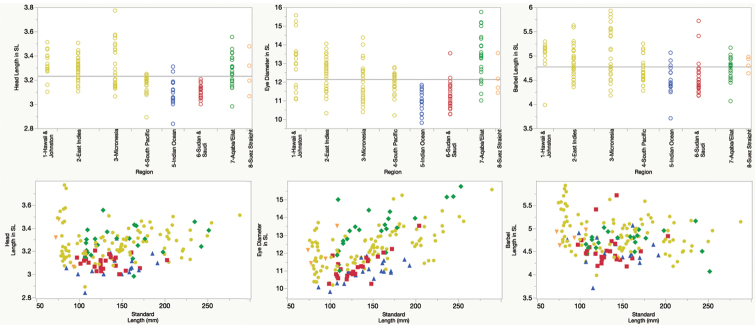
Comparison of head length, eye diameter, and barbel length in SL among regions and against SL in *Mulloidichthys flavolineatus flavolineatus* and *Mulloidichthys flavolineatus flavicaudus* subsp. n. Below, measurements against SL. These colors identify the region of origin of each individual following the scheme in the upper panel. These are the same data as in Table 3.

Meristics are provided in Tables 1 & 2 and measurements as % of SL in Table 4 and Fig. 14. Below, morphometric ratios are given as ratios of SL for the holotype and in parentheses for selected paratypes (n=7), except where indicated.

**Table 1.** Lateral-line scale counts of *Mulloidichthys flavolineatus* subspp. In bold, counts for the holotype of *Mulloidichthys flavolineatus flavicaudus* subsp. n.

		37	38	39	40	mean
*Mulloidichthys flavolineatus flavicaudus* subsp. n.	Gulf of Aqaba	12	10	1		37.5
	Red Sea off Sudan & Saudi Arabia	**18**	5			37.2
†	Maldives	2	4			37.7
*Mulloidichthys flavolineatus flavolineatus*	Islands of Western Indian Ocean ‡	5	5	4		37.9
	East Indies §	4	24	5		38.0
	Islands of Oceania (except Hawai‘i) |	3	26	7		38.1
	Hawaiian Islands & Johnston Atoll	2	16	6	1	38.2

† Both subspecies may overlap and interbreed in Maldives

‡ Chagos Archipelago and Mauritius

§ Indonesia, Papua New Guinea, Philippines and Solomon Islands

| Wake, Minami-Tori Shima, Mariana Islands, Marquesas Islands, Phoenix Islands, Samoa Islands and Rapa

**Table 2.** Total gill-raker counts of *Mulloidichthys flavolineatus*. In bold, counts for the holotype of *Mulloidichthys flavolineatus flavicaudus* subsp. n.

		25	26	27	28	29	30	mean
*Mulloidichthys flavolineatus flavicaudus* subsp. n.	Gulf of Aqaba	3	6	8	2	4		26.9
	Red Sea off Sudan & Saudi Arabia	4	**5**	11	2			26.5
†	Maldives		1	3	2	0		27.2
*Mulloidichthys flavolineatus flavolineatus*	Islands of Western Indian Ocean ‡			4	2	2		27.8
	East Indies §		3	11	12	7		27.7
	Islands of Oceania (except Hawai‘i) |			12	18	11	3	28.4
	Hawaiian Islands & Johnston Atoll			2	3	10	3	28.8

† Both subspecies may overlap and interbreed in Maldives

‡ Chagos Archipelago and Mauritius

§ Indonesia, Papua New Guinea, Philippines and Solomon Islands

| Wake, Minami-Tori Shima, Mariana Islands, Marquesas Islands, Phoenix Islands, Samoa Islands and Rapa

Body elongate, its depth at first dorsal-fin origin 4.1 (4.2–4.5), and maximum width 6.7 (6.5–7.3), head length 3.2 (3.0–3.3, n=27), snout length 6.9 (6.8–7.7), orbit diameter 13.0 (10.3–13.5, n=27), barbel length 4.5 (4.2–5.1, n=27), caudal-peduncle length 4.8 (4.6–5.1), caudal-peduncle depth 11.6 (11.4–12.0), pelvic-fin length 4.9 (4.7–5.3), pectoral-fin length 4.8 (4.7–5.1), longest anal ray 7.5 (7.2–7.7), longest dorsal spine 4.8 (4.6–5.1), longest dorsal ray 7.2 (7.2–7.8).

Mouth small, maxilla not reaching a vertical at front of orbit, upper-jaw length 12.3 (12.2–13.9) in SL; jaws with small conical teeth, in two rows with teeth more irregularly placed between both rows; no teeth on the vomer and palatines; anterior nostril small, elliptical, two-thirds eye diameter in front of eye; posterior nostril small, elliptical, at dorsoanterior corner of orbit; opercular spine flat, at mid-eye height.

Scales very finely ctenoid; head fully scaled; scales on the base of caudal fin, other fins without scales; dorsal fin behind the vertical at fourth lateral line scale, origin of second dorsal above 18th (17th in some paratypes) scale. Pored scales on lateral line with many branching tubules.

#### Color.

Color in life silvery white to yellowish, slightly darker over lateral line; margin of each scale on upper half of body darker than scale. Yellow stripe on side of body at level of eye, from posterior margin of orbit to caudal-fin base, bordered by a narrow whitish stripe (stripe sometimes slightly blue); the stripe usually containing a black spot above posterior part of pectoral fins (under the first dorsal fin), sometimes faint due to fading, stripe anterior to spot occasionally indistinct; barbels white; dorsal fins usually transparent, sometimes first dorsal fin with yellowish tinge; pectoral, anal, and pelvic fins whitish, translucent; caudal fin yellowish or yellow. Color when fresh often pink and all fins yellow. Uniformly creamy white in preservative.

#### Etymology.

*Mulloidichthys flavolineatus flavicaudus* subsp. n. is named in reference to the yellow color of the caudal fin, in contrast to the whitish gray color of the caudal fin of *Mulloidichthys flavolineatus flavolineatus*.

#### Distribution.

*Mulloidichthys flavolineatus flavicaudus* subsp. n. is restricted to the NW Indian Ocean biogeographic province, where it ranges from various locations in the Red Sea (including the Gulf of Aqaba), the Gulf of Tadjoura, the Gulf of Aden, and Socotra (Fig. 9). *Mulloidichthys flavolineatus flavicaudus* subsp. n. has extended its range to Oman (Fig. 11) and probably to the Maldives (Fig. 12), where it has encountered the western distribution of *Mulloidichthys flavolineatus flavolineatus*. Underwater photographs of fish with yellow and gray caudal fins suggest overlap and interbreeding by the two subspecies. Carpenter et al. (1997) included *Mulloidichthys flavolineatus* in their catalog of fishes of the Arabian Gulf. They did not cite any voucher specimens, and the photo they used is from Mauritius.

#### Remarks.

The population of *Mulloidichthys flavolineatus flavicaudus* subsp. n. in the Gulf of Aqaba differs from that in the Red Sea proper by having smaller eyes (11.0–15.8 in SL) and shorter head (3.0–3.6) (Tables 1, 2 and 3). It occasionally also has higher lateral-line scales counts (37–38, occasionally 39) and higher gill-raker counts (25–29).

**Table 3.** Comparison of Head Length, Eye Diameter, and Barbel Length in subspecies of *Mulloidichthys flavolineatus*. Ranges and mean values (in brackets) are given for each ratio.

	Locality	Standard length (mm) and number of specimens	Head length in standard length	Eye diameter in standard length	Barbel length in standard length
*Mulloidichthys flavolineatus flavicaudus* subsp. n.	Gulf of Aqaba	107–252 (n=23)	3.0–3.6 (3.3)	11.0–15.8 (13.4)	4.1–5.2 (4.7)
	Red Sea ‡	97.5–203 (n=28)	3.0–3.2 (3.1)	10.2–13.5 (11.3)	4.2–4.8 (4.5)
†	Maldives	85.5–144 (n=6)	2.8–3.3 (3.2)	10.1–11.8 (10.7)	3.7–4.8 (4.4)
*Mulloidichthys flavolineatus flavolineatus*	Indian Ocean §	120–192 (n=12)	3.0–3.3 (3.1)	10.5–11.7 (11.0)	4.2–5.1 (4.5)
	East Indies |	98–255 (n=34)	3.1–3.5 (3.3)	10.3–14.0 (12.5)	4.3–5.6 (4.9)
	Micronesia ¶	75–230 (n=26)	3.1–3.8 (3.3)	10.4–14.4 (11.9)	4.2–5.9 (5.0)
	South Pacific #	81–198 (n=26)	2.9–3.4 (3.2)	10.2–12.8 (11.9)	4.3–5.2 (4.7)
	Hawaiian Is. ††	83–288 (n=16)	3.1–3.7 (3.3)	10.4–15.6 (12.9)	4.0–6.0 (5.1)

† Both subspecies may overlap and interbreed in Maldives

‡ Off Sudan and Saudi Arabia

§ Chagos Archipelago and Mauritius

| Indonesia, Papua New Guinea, Philippines, and Solomon Islands

¶ Wake, Minami-Tori Shima and Mariana Islands

# Marquesas Islands, Phoenix Islands, Samoa Islands and Rapa

†† Including Johnston Atoll

**Table 4.** Proportional measurements of type specimens of *Mulloidichthys flavolineatus flavicaudus* subsp. n. and of comparative material of *Mulloidichthys flavolineatus flavolineatus* as percentages of the standard length.

	***Mulloidichthys flavolineatus flavicaudus* subsp. n.**								***Mulloidichthys flavolineatus flavolineatus***			
	**Sudan**								**Mauritius**			**Johnston Atoll**
	**Holotype**								**Neotype**			
	SMF 35486	SMF 24818-1	SMF 24818-2	SMF 24818-3	SMF 24818-4	SMF 24818-5	SMF 24818-6	USNM 221124	BPBM 20135	BPBM 41252–1	BPBM 41252–2	BPBM 7520
Standard length (mm)	142	125	120	118	111	108	106	181	162	120	135	166
Body depth	24.3	23.1	23.2	24.1	22.8	22.1	22.2	22.1	22.2	22.1	22.0	25.1
Body width	14.9	14.9	15.3	14.4	14.5	13.6	13.9	15.1	13.1	12.3	9.6	14.2
Head length	31.3	33.2	32.6	32.3	31.9	32.1	30.9	32.1	30.6	31.5	32.2	33.2
Snout length	14.4	14.4	13.9	13.5	14.7	13.2	13	14.4	14.5	13.1	13.5	15.2
Orbit diameter	7.7	9.3	9.5	9.0	8.8	9.2	7.7	8.6	9.2	8.5	8.6	9.3
Interorbital width	7.8	7.5	7.2	7.4	7.8	7.0	7.0	7.5	9.0	7.6	6.9	8.0
Upper-jaw length	8.1	7.0	8.0	8.0	7.8	7.2	7.6	8.2	8.3	8.4	8.1	7.8
Barbel length	22.3	23.4	24.0	22.5	21.8	22.5	–	21.0	19.8	20.8	22.2	20.0
Caudal-peduncle least depth	8.6	8.6	8.3	8.6	8.5	8.4	8.7	8.8	9.2	8.8	9.2	9.2
Caudal-peduncle length	24.3	22.4	20.1	23.0	23.1	24.9	21.8	25.2	23.3	30.5	20.3	23.4
Snout to origin of first dorsal fin	41.1	39.2	39.6	39.3	40.4	37.3	27.3	38.6	40.6	38.9	40.1	42.0
Snout to origin of second dorsal fin	68.1	67.0	66.6	67.3	66.5	63.8	65.1	65.5	65.9	62.8	63.2	68.8
Preanal-fin length	69.4	65.6	67.5	68.9	66.5	66.4	65.7	67.7	67.0	64.8	6.7	67.4
Prepelvic-fin length	33.2	33.2	35.2	34.9	32.8	34.3	33.0	33.5	32.9	31.3	31.8	32.7
Second dorsal-fin base	12.8	12.5	11.7	12.3	12.2	11.5	12.1	13.1	11.8	10.8	11.1	12.4
Anal-fin base	9.9	10.5	9.1	10.6	9.6	9.9	10.7	9.7	9.0	10.4	9.4	10.4
First dorsal-fin base	16.7	18.8	19.9	21.8	17.6	16.6	17.4	17.2	15.3	17.3	16.0	19.0
Pectoral-fin base	4.9	5.1	5.2	5.2	5.7	4.7	4.9	5.6	5.0	5.2	4.8	5.4
Longest dorsal spine	20.8	19.9	21.3	21.8	19.7	18.7	19.0	21.3	20.6	20.2	21.2	20.1
Longest dorsal ray	14.6	14.1	14.1	13.4	14.3	14.5	13.2	13.4	14.3	15.4	15.2	14.3
Longest anal ray	13.9	13.4	13.5	13.7	13.2	12.9	13.6	13.8	13.3	14.7	14.4	13.7
Caudal-fin length	28.1	27.1	26.8	–	26.6	24.9	25.0	–	26.3	27.5	28.6	25.9
Caudal concavity	19.1	18.1	19.1	–	16.4	16.9	16.5	–	16.7	19.7	20.6	16.8
Pectoral-fin length	20.8	20.3	20.6	21.5	21.2	19.9	20.7	19.8	20.0	21.3	–	21.7
Pelvic-fin length	20.6	20.3	20.4	21.0	20.1	19.0	21.1	19.8	20.3	22.1	21.1	21.0

* Two specimens of BPBM 41252 (103 and 111 mm), from Mauritius were damaged and not included in the table.

#### Comparisons.

*Mulloidichthys flavolineatus flavicaudus* subsp. n. differs from its nominal subspecies *Mulloidichthys flavolineatus flavolineatus* in having 25–28 (usually 26 or 27) gill-raker counts (26–30, usually 27–29, in *Mulloidichthys flavolineatus flavolineatus*), usually 37–38 lateral-line scales (37–40 in *Mulloidichthys flavolineatus flavolineatus*) and a yellow caudal fin (white to light gray in *Mulloidichthys flavolineatus flavolineatus*). Also, the eyes are smaller in *Mulloidichthys flavolineatus flavicaudus* subsp. n. (10.3–13.5 in SL) than in *Mulloidichthys flavolineatus flavolineatus* (9.8–15.6 in SL).

### Mulloidichthys flavolineatus flavolineatus

Taxon classificationAnimaliaPerciformesMullidae

(Lacepède, 1801)

Yellowstripe Goatfish

Figures 1 , 2 , 3 , 5 , 6 , 14 ; Tables 1 , 2 , 3 , 4

Mullus flavolineatus Lacepède, 1801: 384, 406 (locality unknown, no types known).
Mulloidichthys flavolineatus (Lacepède 1801): Randall and Anderson 1993: 20 (Maldives); Randall et al. 1997: 208 (Great Barrier Reef); Kuiter 1998: 117 (Maldives: in part, upper photo); Anderson 2005: 57 (Maldives); Allen et al. 2007: 122 (Christmas Island); Okamura and Okamoto 1997: 373 (Japan); Randall 2007: 260 (Hawaiian Islands); Myers 1999: 159, Pl. 74H (Micronesia); Matsunuma et al. 2011: 142 (Malaysia); Uiblein 2011: 59 & 69, Pl. 1 (description, color images); Allen and Erdmann 2012: 504 (Philippines).

#### Neotype.

BPBM 20135, 162 mm SL, Indian Ocean, Mauritius, East Coast, Oyster Bay (19°43'S; 63°21'E), 1 November 1973, coll. J.E. Randall.

#### Non-type material.

**Hawaiian Islands**: BPBM 28726, 83 mm SL, Kona Coast, South Kohala; BPBM 4087, 288 mm SL, Laysan; BPBM 4086, 180 mm SL, Laysan; BPBM 4088, 2: 139–230 mm SL, Lisiansky; BPBM 25457, 130 mm SL, O‘ahu, Wai‘anae coast; BPBM 25674, 175 mm SL, O‘ahu; BPBM 1749, 183 mm SL, O‘ahu; BPBM 1750, 173 mm SL, O‘ahu; BPBM 15308, 152 mm SL, Midway Atoll; BPBM 25517, 119 mm SL, Midway Atoll; USNM 147073, 158 mm SL, Midway Atoll. **Johnston Atoll**: BPBM 4090, 85 mm SL; BPBM 4091, 93 mm SL; BPBM 7520, 166 mm SL; **Philippines**: USNM 327877, 107 mm SL, Sorsogon, Gubat Bay; USNM 405724, 209 mm SL, W Luzon, Port Matalvi; USNM 147062, 222 mm SL, Mindoro, Varadero Bay; USNM 147066, 169 mm SL, W Luzon, Zambales; USNM 322272, 3: 138-155 mm SL, Babuyan, Maybag Island; USNM 147069, 2: 171–176 mm SL, Batangas, Maricaban; USNM 84231, 128 mm SL, Mindanao, Zamboanga; USNM 84232, 139 mm SL, Mindanao, Zamboanga; USNM 147070, 164 mm SL, Palawan, Candaraman; USNM 147072, 145 mm SL, Tulayan Island, Jolo; USNM 145294, 2: 98–100 mm SL; USNM 147065, 231 mm SL, Sulu, Siasi Island; USNM 147076, 135 mm SL, Sulu, Simaluc Island, Tawi Tawi. **Indonesia**: USNM 147067, 221 mm SL, Moluccas, Bouru Island; USNM 147064, 3: 195–205 mm SL, Moluccas, Makian I; USNM 405723, 200 mm SL, Moluccas, Makian Island; USNM 267514, 2: 102–126 mm SL, Mentawai Islands, Pulau Siburu; USNM 267503, 2: 155–156 mm SL, Mentawai Islands, Pulau Siburu; USNM 147058, 203 mm SL, Sulawesi, Talisse Island; USNM 87989, 255 mm SL, Sumatra, Poeloe Toekus; USNM 75887, 250 mm SL, Borneo, Tandjoeng, Setebah. **Cocos-Keeling**: SU 35630, 200 mm SL, Cocos-Keeling I. **Papua New Guinea**: USNM 267499, 203 mm SL, Trobriand Kuia Islands; USNM 267515, 2: 116–120 SL, New Britain, Rabaul. **Solomon Islands**: USNM 382371, 205 mm SL, Santa Cruz Islands. **Micronesia**: BPBM 77, 8: 75–230 mm SL, Guam; BPBM 4089, 3: 209–220 mm SL, Wake Island; BPBM 24628, 12: 79–160 mm SL, Chuuk, Puluwat Atoll. **Japan**: BPBM 7086, 2: 88–108 mm SL, Minami-Tori Shima; BPBM 7087, 210 mm SL, Minami-Tori Shima. **South Pacific**: BPBM 27868, 5: 81–119 mm SL, Samoan Islands, Tutuila Island; BPBM 27906, 3: 84–107 mm SL, Samoan Islands, Tutuila Island; BPBM 15299, 16: 81–159 mm SL, Phoenix Islands, Orona Atoll; BPBM 12937, 165 mm SL, Rapa; BPBM 2136, 198 mm SL, Marquesas Islands, Nuku Hiva. **Western Indian Ocean**: USNM 229036, 9: 129–192 mm SL, Chagos Archipelago, Salomon Atoll; CAS 237312, 2: 137–144 mm SL, Maldives, Faafu Atoll; BPBM 34673, 2: 107–115 mm SL, Maldives, N Malé Atoll; CAS 35383, 2: 85.5–142 mm SL, Maldives, Malé Atoll; BPBM 41252, 2: 120–135 mm SL, Mauritius, Oyster Bay.

#### Diagnosis.

Body elongate, the depth at first dorsal-fin origin 4.0–4.6 in SL; head moderately compressed, the length 2.9–3.8 in SL; snout long, slightly blunt anteriorly. Barbels usually not reaching a vertical at posterior margin of preopercle, their length 3.7–6.0 in SL. Eye diameter 10.1–15.6 in SL. Pectoral-fin rays 16–18. Gill-raker counts 27–29 (rarely 26 or 30); lateral-line scales 37–40 (usually 38). Caudal fin varying from usually white or light gray to occasionally yellowish or yellow.

#### Color.

Silvery white to yellowish, slightly darker over lateral line, margins of each scale on upper half of body darker than scale. Yellow stripe on side of body at level of eye, beginning from posterior margin of orbit and ending at caudal-fin base, bordered by two whitish narrow stripes (sometimes slightly blue); the stripe usually containing a black spot above posterior part of pectoral fins (under the first dorsal fin), sometimes faint due to fading, stripe anterior to spot occasionally indistinct; barbels white; dorsal fins usually transparent, sometimes first dorsal fin with yellowish tinge; pectoral, anal, and pelvic fins whitish, translucent; caudal fin varying from usually white or light gray to occasionally yellowish or yellow. Sometimes body color pattern of broad irregular red-brown bars, especially at night. When fresh, body color can turn pink and all fins yellow. Uniformly creamy white in preservative.

#### Distribution.

**Figure 15.**
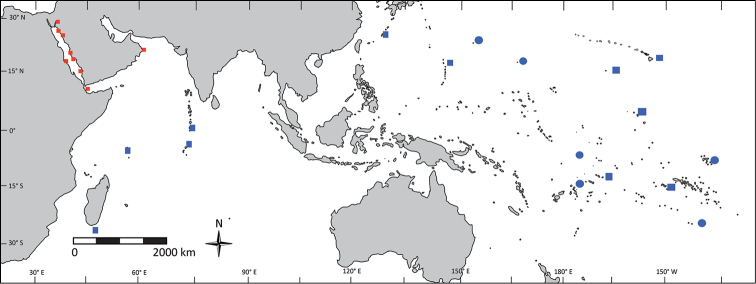
Distribution map of *Mulloidichthys flavolineatus* surveyed in this study. Red symbols denote locations of specimens of *Mulloidichthys flavolineatus flavicaudus* subsp. n. and blue symbols denote locations of specimens of *Mulloidichthys flavolineatus flavolineatus*. Squares indicate locations included in the genetic surveys. Circles indicate locations of specimens for which only morphological analyses were carried out.

*Mulloidichthys flavolineatus flavolineatus* is wide-ranging from East Africa north to the Maldives and Chagos Archipelago and east to the Hawaiian, Marquesas and Pitcairn Islands, north to the Ryukyu and Bonin Islands and south to Lord Howe Island, New Caledonia and Rapa Island (Randall 2002, Uiblein 2011) (Fig. 15).

#### Genetics.

**Figure 16.**
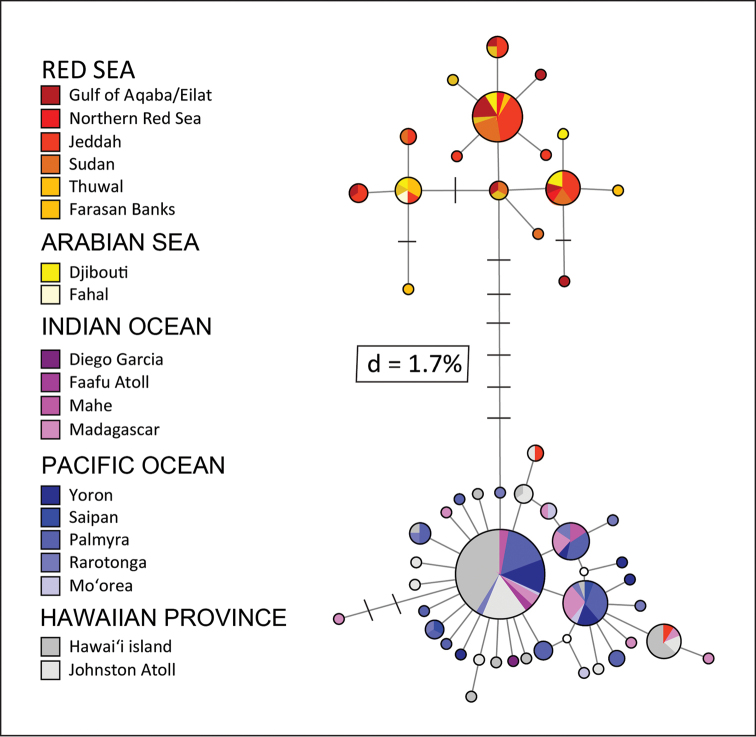
Median-joining haplotype network based on mitochondrial *cytochrome b* sequence data (715 bp) from 217 *Mulloidichthys flavolineatus* individuals sampled across the Red Sea, Arabian Sea, Indian Ocean and Pacific Ocean. Each circle represents a haplotype, with size proportional to its total frequency. Branches separated by black crossbars represent a single nucleotide change, whereas open circles indicate unsampled haplotypes; colors indicate collection location as in the embedded key. The network depicts two distinct clades separated by seven mutational steps (corrected sequence divergence, d = 1.7%; Kimura 1980) (From Fernandez-Silva et al. 2015).

The parsimony-based haplotype networks constructed with mtDNA *cytb* sequences from 217 *Mulloidichthys flavolineatus* specimens revealed a separation between individuals from the NW Indian Ocean (including the Red Sea, the Gulf of Aden and Oman) and individuals in the rest of the Indian Ocean and the Pacific Ocean (Fig. 16). Corrected genetic distance was 1.7%, with seven diagnostic mutations (Fernandez-Silva et al. 2015).

**Figure 17.**
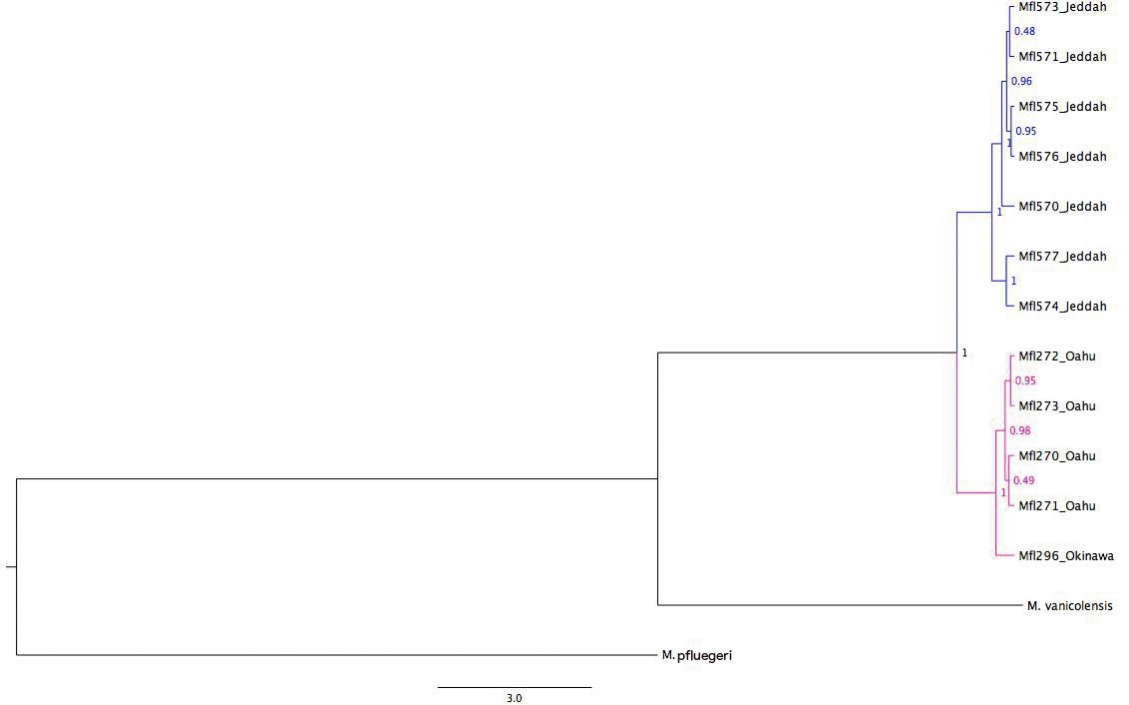
Bayesian-inference based phylogenetic tree showing relationships among mtDNA concatenated haplotypes of segments of the *cytb* and *ATPase-8* & *ATPase-6* genes from seven individuals of *Mulloidichthys flavolineatus flavicaudus* subsp. n. from the Red Sea (Jeddah), five individuals of *Mulloidichthys flavolineatus flavolineatus* from the Pacific (O‘ahu in Hawai‘i and Okinawa) and two *Mulloidichthys* spp. as outgroups. The nodes show posterior probabilities. Branch lengths are according to estimated divergence time (note that the branch leading to *Mulloidichthys pfluegeri* was reduced by 50%).

We obtained a concatenated alignment of a 715-bp segment of the *cytb* gene and a 731-bp segment of the *ATPase-8* and *ATPase-6* genes of the mitochondrial genome from seven individuals from the Red Sea (Jeddah) and five from the Pacific (Hawai‘i and Okinawa). Phylogenetic reconstructions based on Bayesian inference (Fig. 17) revealed a genetic break and the presence of two well-supported monophyletic clades (posterior probability = 1): one with sequences from the Red Sea and one with the haplotypes from the Pacific. Reconstructions based on the Maximum-Likelihood and Neighbor-Joining methods were in agreement with this topology but clades had lower statistical support (results not shown).

## Discussion

Higher gill-raker and lateral-line counts, smaller eyes and stable yellow coloration of the caudal fin in *Mulloidichthys flavolineatus* from the Red Sea are characters in alignment with the genetic isolation of a mitochondrial lineage in the NW Indian Ocean biogeographic province (as per Kulbicki et al. 2013) and support the subspecies designation of *Mulloidichthys flavolineatus flavicaudus* subsp. n.

Some ichthyologists, notably Gill (1999), have questioned the validity of subspecies in marine fishes, especially in reference to wide-ranging Indo-Pacific species. One could argue that the existence of subspecies should be demonstrated by intermediates between two isolated populations before they could be labeled as subspecies. Divisions of populations into two or more populations have resulted from the change in sea level caused by the variation in the size of the polar ice caps. The Indian Ocean was isolated from the Pacific, and the Red Sea from the Indian Ocean when the ice caps were very large. We assume that the yellow-tailed population of *Mulloidichthys flavolineatus* arose as a subspecies when the Red Sea was isolated, approximately half a million years ago assuming a molecular clock of 2% divergence per million years (as per Bowen et al. 2001). This population persisted in isolation through several Pleistocene glacial cycles (Fernandez-Silva et al. 2015) and over time extended out to Socotra, Oman and possibly Maldives, where it entered into secondary contact with the Indo-Pacific population. In the second author’s book *Coastal Fishes of Oman* (Randall 1995), a single individual of *Mulloidichthys flavolineatus* is illustrated as Figure 620. It has a yellowish caudal fin. He wrote in the brief species account, “fins whitish, the caudal fin often yellowish.” The underwater photograph of *Mulloidichthys flavolineatus* of Fig. 11 taken on the south coast of Oman shows caudal fins varying from pale greenish gray (the green part from the sea color) to a few all yellow. This photograph suggests that the two subspecies of *Mulloidichthys flavolineatus* may overlap and interbreed, hypotheses to be confirmed with genetic methods. The geographic extension of the yellow-tailed subspecies in the understudied Western Indian Ocean warrants further investigation.

Notably, the age of split of the *Mulloidichthys flavolineatus* subspecies is older than the radiation that gave rise to *Mulloidichthys vanicolensis*, *Mulloidichthys mimicus*, *Mulloidichthys dentatus* (Gill, 1862) and *Mulloidichthys martinicus* (Cuvier, 1829) less than 350,000 years ago (unpublished results).

It is remarkable that individuals of *Mulloidichthys flavolineatus flavicaudus* subsp. n. from the Gulf of Aqaba have consistently smaller eyes, longer head, and longer barbels than fish from the Red Sea proper (Fig. 14). Pelvic fins are also shorter in the Gulf of Aqaba (mean length in SL = 5.17) than in the rest of the Red Sea (4.40 in SL). However, both populations extensively share *cytb* haplotypes and the analyses of haplotype frequencies do not support genetic differentiation, although this comparison is based on mitochondrial markers only (Fernandez-Silva et al. 2015). In the northern tip of the Gulf of Aqaba, *Mulloidichthys flavolineatus flavicaudus* was among the 11 most common species on the shallow sandy habitat, but all specimens were juveniles or subadults (maximum length: 15 cm TL) (Golani 1993; Golani and Lerner 2007). The Gulf of Aqaba has remarkably high endemism. Twenty-six species of fishes, including the goatfish *Upeneus davidaromi*, are known to the Gulf of Aqaba only (Table 5). Although further research may result in range extensions for some of these fishes to the Northern Red Sea, the number of endemics is very high for an area of only 160 × 24 km. Environmental differences could explain this isolation. The Gulf of Aqaba is much deeper (1850 m) than the Red Sea to the south, and seawater temperature is considerably lower (20–27°C) and salinity higher (40–41‰) than in the Red Sea proper (25–31°C; 37–41‰) (Oren 1962). Moreover, the Gulf may have acted as a glacial refuge for reef fauna during Pleistocene low sea level stands, when most of the Red Sea was too saline for coral reef development. Geological and paleoclimatic research suggest that during these periods the Gulf of Aqaba, owing to rainfall and fluvial intake, maintained lower salinity levels and that environmental conditions were favorable to sustain coral reefs and associated fauna (DiBattista et al. 2016). Therefore, the Gulf of Aqaba served as a refuge for marine life from the harsh marine environment to the south. Parapatric speciation processes reinforced by selection may account for the elevated endemism in the region (Golani 1993; Por 2008; Tikochinski et al. 2013).

**Table 5.** Endemic fishes of the Gulf of Aqaba.

Endemic fishes of the Gulf of Aqaba	Remarks
*Amblyeleotris neglecta* Jaafar & Randall, 2009	
*Cabillus nigrostigmus* Kovačić & Bogorodsky, 2013	Known from Sharm el Moya, close to the entrance of the Gulf of Aqaba
*Callionymus profundus* Fricke & Golani, 2013	Deep-water species
*Chromis pelloura* Randall & Allen, 1982	
*Cirrhilabrus blatteus* Springer & Randall, 1974	
*Evoxymetapon moricheni* Fricke, Golani & Appelbaum–Golani, 2014	
*Gymnapogon melanogaster* Gon & Golani, 2002	
*Gymnothorax baranesi* Smith, Brokovich & Einbinder, 2008	
*Hetereleotris psammophila* Kovačić & Bogorodsky, 2014	Recently photographed at Safaga
*Heteronarce bentuviai* (Baranes & Randall, 1989)	
*Limnichthys marisrubri* Fricke & Golani, 2012	
*Myxomyrophis longirostris* Hibino, Kimura & Golani, 2014	
*Paragunnellichthys springeri* Dawson, 1970	Formally endemic to Gulf of Aqaba, known from Sharm el Moya, close to the entrance
*Parascolopsis baranesi* Russell & Golani 1993	
*Pseudogramma megamyctera* Randall & Baldwin, 1997	Reported from West Papua (Allen and Erdmann 2012); a record probably represented by a similar undescribed species
*Scorpaenodes steinitzi* Klausewitz & Fröiland, 1970	A specimen identified as *Scorpaenodes steinitzi* collected from Djibouti, but no voucher available for confirmation
*Stalix davidsheni* Klausewitz, 1985	
*Suculentophichthus nasus* Fricke, Golani & Appelbaum–Golani, 2015	
*Symphysanodon disii* Khalaf & Krupp, 2008	
*Syngnathus safina* Paulus, 1992	
*Thamnaconus erythraeensis* Bauchot & Maugé, 1978	
*Tomiyamichthys dorsostigma* Bogorodsky, Kovačić & Randall, 2011	
*Upeneus davidaromi* Golani, 2001	
*Uropterygius genie* Randall & Golani, 1995	Known at Ras Mohammed, close to the entrance of the Gulf of Aqaba
*Uropterygius golanii* McCosker & Smith, 1997	Known at Ras Mohammed, close to the entrance of the Gulf of Aqaba
*Vanderhorstia opercularis* Randall, 2007	

Our range-wide phylogeographic survey of *Mulloidichthys flavolineatus* (Fernandez-Silva et al. 2015) indicated the genetic isolation of the Hawaiian population (including Johnston Atoll) from the remainder of the Indo-Pacific. Uiblein (2011) indicates that Pacific Ocean *Mulloidichthys flavolineatus* have shorter barbels than those in the Indian Ocean, but he includes the Hawaiian Islands with the rest of the Pacific in this study. We found the Hawaiian population has shorter barbels, shorter head, smaller eyes, higher gill-raker counts, and higher lateral-line scale counts than all other populations examined, and that there is a range of variation as we move from Hawai‘i to other islands of Oceania, the West Indies, the Western Indian Ocean, and the Red Sea (Tables 1–3, and Fig. 14).

Fernandez-Silva et al. (2015) listed four Red Sea endemic species of Mullidae: *Parupeneus forsskali* (Fourmanoir & Guézé, 1976), *Upeneus davidaromi*, *Upeneus niebuhri* Guézé, 1976, and *Upeneus pori* Ben-Tuvia & Golani, 1989, but inclusion of the latter to the Red Sea endemics is a mistake, as this species is also reported from Oman, Madagascar and South Africa.

## Supplementary Material

XML Treatment for Mulloidichthys flavolineatus flavicaudus

XML Treatment for Mulloidichthys flavolineatus flavolineatus
